# Genito-Urinary and Syphilitic Diseases

**Published:** 1900-03

**Authors:** Byron H. Daggett

**Affiliations:** Buffalo, N. Y.


### GENITO-URINARY AND SYPHILITIC DISEASES.

CONDUCTED BY BYRON H. DAGGETT, M. D., Buffalo, N. Y.

EXCRETION IN THE TREATMENT OF ACUTE INFECTIOUS DISEASES.

H. A. McCOLLUM (*Philadelphia Medical Journal*, January 13, 1900,) says that purgation, diaphoresis, emesis and diuresis have a much wider range of usefulness than is generally recognised. The clinical physiology of excretion is obscure. The amount of lymph in the human body exceeds that of the blood three or four times, and the importance of this primeval circulation can scarcely be overestimated. How lymph is enriched and purified is a mooted question, but it is agreed that diminution in the quantity of blood and increase of vascular area will force it into the blood current and increased capillary pressure drives more blood plasma into the lymph circulation. All the salines, such as the soda, magnesia and potassium compounds cause an interchange between the blood and lymph and are called lymphagogs. This interchange occurs between the lymph spaces and the blood capillaries.

The thoracic duct supplies but a fractional part of the lymph to the blood when bleeding. The last flow is more dilute than the first, even though the thoracic duct be ligated. The varying pressure of arterial capillaries and venous capillaries cause tissue irrigation. The tissue spaces are reservoirs anatomically independent of both blood and lymph capillaries, yet they supply or interchange with both systems. Tissue oxidation produces tissue activity and then follows excretion.

Shafer, speaking on the internal secretion of glands, says: "In one sense all the tissues and organs of the body form internal secretion, for they all pass into the blood materials which have been formed as products of metabolism.'' Internal secretions give to the lymph not only waste products, but other products that are to be of use to the system.

The internal excretions supply defensive proteids (antitoxin) in all probability and their function in duplex. Research as shown that the action of blood-letting, emesis, cold-baths, purging, and the like, support this view.

The vital activities of the tissues promote internal excretion and internal excretion in turn protects the body tissues, especially the central nervous system from the baneful effects of toxins. ''The decisive battle-ground in acute infectious diseases is in the central nervous system, when the rhythmically acting vasomotor, cardiac and respiratory centers must be preserved intact from harmful toxins.'' A saline purge acts as an internal excretion agent and secondly as a purgative.

T. Landor Brunton says that a purge acts as an internal excretion agent and as a promotor of serum therapeutics.

Maurel and Robin allege that blood-letting, blistering and emetics act much alike in increasing oxidation and leucocytosis. The cold bath in fever acts by increasing internal respiration or oxidation and metabolic activity.

Venesection, purging, emesis, diaphoresis, diuresis, and the like, are most valuable auto-serum therapeutic agents. These valuable forms of treatment have fallen into disfavor; the newer medicine is bringing them to the front again.

---

REMOVAL OF FOREIGN BODIES FROM THE NOSE AND EAR.—Starrock (*Brit. Med. Jour.*, December 30, 1899,) recommends the following means of procedure: The presence and approximate situation of the foreign body having been ascertained, a piece of india rubber tubing rather less in diameter than an ordinary lead pencil, varying in length from one to three inches, and attached to the nozzle of a brass syringe, is introduced into the nostril or meatus, as the case may be, and brought into contact with the foreign body. The piston of the syringe is then pulled out for a sufficient distance to create a vacuum in the tubing, and thus draw the foreign body into, or against, its free end. The syringe is then withdrawn and with it the foreign body attached to the tubing. In some cases it has been found advantageous to dip the tubing into glycerin before inserting, in order to diminish the chances of air entering between the tubing and the foreign body.—*Medical Record.*

